# Association between serum insulin levels and ultrasound-defined nonalcoholic fatty liver disease: A cross-sectional study based on NHANES 2017–2020

**DOI:** 10.1097/MD.0000000000047019

**Published:** 2026-01-16

**Authors:** Mao Li, Shunhua Qiu, Min Xiang

**Affiliations:** a Department of Ultrasound Medicine, The Third People’s Hospital of Zigong, Zigong, Sichuan Province, China; b Department of Clinical Laboratory, The Third People’s Hospital of Zigong, Zigong, Sichuan Province, China.

**Keywords:** controlled attenuation parameter, epidemiological study, NHANES, nonalcoholic fatty liver disease, serum insulin

## Abstract

Nonalcoholic fatty liver disease (NAFLD) is one of the most common chronic liver diseases worldwide. It is closely linked to various metabolic abnormalities. Serum insulin level, an important compensatory marker of insulin resistance, plays a key role in the development and progression of NAFLD. However, large-scale studies evaluating the association between serum insulin and NAFLD based on imaging-defined standards, such as the controlled attenuation parameter (CAP), are still limited. This study analyzed data from National Health and Nutrition Examination Survey 2017–2020. A total of 2791 eligible United States adults were included. Liver CAP values were measured using transient elastography, and fatty liver was defined as CAP ≥ 248 dB/m. Fasting serum insulin levels were also measured. Multivariable weighted logistic regression, restricted cubic spline analysis, and receiver operating characteristic curve analysis were used. These methods assessed the association between serum insulin levels and ultrasound-defined NAFLD, adjusting for demographic and metabolic confounders. After comprehensive adjustment for covariates, serum insulin levels treated as a continuous variable were significantly associated with increased odds of having NAFLD (odds ratio [OR] = 1.04; 95% confidence interval [CI]: 1.01–1.07; *P* = .014). Quartile-based analysis indicated that individuals in the highest insulin group (Q4) had significantly higher odds of having NAFLD compared with those in the lowest insulin group (Q1; OR = 3.61; 95% CI 1.84–7.08; *P* = .006). Restricted cubic spline analysis revealed a significant positive nonlinear relationship between serum insulin levels and NAFLD (*P* for non-linearity < .05). Receiver operating characteristic curve analysis demonstrated that serum insulin had good discriminative ability for predicting NAFLD, with an area under the curve of 0.774 (95% CI: 0.757–0.792). Multiple sensitivity and subgroup analyses further confirmed the robustness of the results. Overall, these findings suggest that serum insulin levels are significantly and positively associated with ultrasound-defined NAFLD. Serum insulin also shows good independent predictive ability. It may serve as an important metabolic biomarker for early screening and risk assessment of fatty liver.

## 1. Introduction

Nonalcoholic fatty liver disease (NAFLD) refers to a group of clinical and imaging syndromes characterized by abnormal fat deposition in the liver parenchyma after excluding significant alcohol consumption and other specific liver diseases.^[1]^ NAFLD has become one of the most common chronic liver diseases worldwide, with a prevalence of approximately 25% in the general population.^[2]^ Although NAFLD is generally considered a benign condition, if not properly managed, it can progress to nonalcoholic steatohepatitis,^[3,4]^ liver fibrosis, and even cirrhosis, eventually leading to liver failure or hepatocellular carcinoma.^[5,6]^ Moreover, NAFLD is closely associated with metabolic syndrome, atherosclerosis, myocardial infarction, and stroke. It is recognized as an important independent risk factor for increased all-cause mortality.^[7–10]^

Imaging plays an increasingly important role in the diagnosis of NAFLD. In particular, the controlled attenuation parameter (CAP) measured by transient elastography has become a noninvasive, quantitative, and sensitive tool for fatty liver screening.^[11]^ CAP can accurately assess hepatic fat content and shows good consistency with liver biopsy results. It is especially suitable for large-scale epidemiological studies and early screening interventions.^[12]^ In the pathogenesis of NAFLD, insulin resistance (IR) is widely regarded as the central pathophysiological basis.^[13]^ IR promotes lipid accumulation in hepatocytes by inhibiting fatty acid oxidation, enhancing hepatic triglyceride synthesis, and increasing glucose output. It can also trigger oxidative stress and inflammatory responses, further facilitating the progression from NAFLD to nonalcoholic steatohepatitis.^[14–16]^ Serum insulin level, as a key compensatory marker of IR, may not only reflect metabolic disorders but also directly contribute to the development and progression of NAFLD.^[17,18]^

Although previous studies have explored the relationship between IR and NAFLD, few large-scale population-based studies have specifically examined the direct association between serum insulin levels and CAP-defined NAFLD. Moreover, the potential nonlinear dose–response relationship and predictive value of serum insulin for NAFLD have not been fully elucidated. Therefore, this study utilized data from the National Health and Nutrition Examination Survey (NHANES) 2017–2020. By combining restricted cubic spline (RCS) and receiver operating characteristic (ROC) analyses, this study aimed to comprehensively evaluate the nonlinear and predictive associations between serum insulin levels and NAFLD. These findings provide novel evidence for the role of insulin as a metabolic biomarker in early detection and risk stratification of NAFLD, beyond traditional IR frameworks.

## 2. Materials and methods

### 2.1. Data source

The data for this study were obtained from the NHANES during the 2017–2020 cycle. NHANES is an ongoing, nationally representative survey conducted by the National Center for Health Statistics using a stratified, multistage probability sampling design. Its primary goal is to assess the health and nutritional status of the United States population. The survey integrates dietary assessments, questionnaire interviews, physical examinations, laboratory testing, and imaging studies. Due to its representative sampling and comprehensive data collection, NHANES is widely used in public health and epidemiological research. The NHANES protocol was approved by the National Center for Health Statistics Research Ethics Review Board, and all participants provided written informed consent. The NHANES datasets are publicly available for download and research use. As this study involved secondary analysis of publicly available, de-identified NHANES data, additional institutional ethical approval was not required.^[19,20]^

### 2.2. Participant selection criteria

The study population included individuals who completed the following 3 types of data collection: liver ultrasound transient elastography with available CAP measurements; fasting serum insulin concentration testing; and demographic information and key metabolic-related covariates. During data cleaning, for categorical variables with <5% missing data, missing values were handled by direct deletion. For categorical variables with more than 5% missing data, missingness was treated as a separate category to maintain data integrity and control bias. For continuous variables with missing data, median imputation was used to maximize the sample size and minimize the influence of outliers.

### 2.3. Assessment of ultrasound-defined NAFLD

This study assessed fatty liver using vibration-controlled transient elastography (VCTE). VCTE is a noninvasive imaging technique that evaluates liver stiffness and fat content by measuring shear wave velocity and ultrasound signal attenuation. The core indicator of VCTE is the CAP, expressed in dB/m. Higher CAP values indicate more severe hepatic fat deposition. When compared with conventional B-mode ultrasound, VCTE offers higher sensitivity and specificity for early detection of fatty liver and provides objective quantitative data. In this study, NAFLD was defined as a CAP value ≥ 248 dB/m, based on previous research.^[21]^ NAFLD was further defined by meeting all the following criteria: CAP value ≥ 248 dB/m; absence of excessive alcohol consumption (≤20 g/d for men and ≤ 10 g/d for women)^[22]^; and no evidence of hepatitis B or C virus infection (negative for hepatitis B core antibody), hepatitis C virus RNA (HCV RNA), and anti-HCV.^[21]^ Participants were classified into the NAFLD group or the non-NAFLD group based on CAP values for further analysis of the association with serum insulin levels.

### 2.4. Measurement of serum insulin

Fasting serum insulin data were obtained from the fasting subsample of the NHANES 2017–2020 cycle. Participants who completed blood collection after 8 to 24 hours of fasting were included. Insulin levels were measured using a two-site immunoenzymometric assay (AIA-PACK IRI) and analyzed with Tosoh AIA automated immunoassay analyzers. All samples were collected at mobile examination centers and transported under cold chain conditions to the University of Missouri-Columbia laboratory for centralized testing. Laboratory quality control procedures complied with the standards of the Clinical Laboratory Improvement Amendments of 1988. Serum insulin concentrations were reported in μU/mL.^[23]^

### 2.5. Covariates

In analyzing the association between serum insulin levels and NAFLD prevalence, multiple covariates were included to control for potential confounding factors. Specifically, the following variables were adjusted for: age, gender (male/female), race/ethnicity (Mexican American/other Hispanic, non-Hispanic White, non-Hispanic Black, and other), education level (less than high school, high school or equivalent, college or above),^[24]^ and marital status (married/living with partner, widowed/divorced/separated, and never married). Other covariates included the poverty income ratio (PIR), smoking status (never smoker, former smoker, and current smoker),^[25]^ presence of hypertension and diabetes, body mass index (BMI), total cholesterol, triglycerides, aspartate aminotransferase (AST), alanine aminotransferase (ALT), and high-sensitivity C-reactive protein (hs-CRP).

### 2.6. Statistical analysis

First, serum insulin levels were divided into 4 quartiles: Q1, Q2, Q3, and Q4. Categorical variables were presented as weighted percentages (%), and group comparisons were performed using weighted chi-square tests. For continuous variables with normal distribution, data were expressed as means ± standard deviations and compared using weighted one-way analysis of variance. For non-normally distributed continuous variables, data were expressed as medians and interquartile ranges, and comparisons were made using the weighted Kruskal-Wallis rank-sum test. Second, to assess the association between serum insulin levels and the prevalence of NAFLD, 3 multivariable weighted logistic regression models were constructed. In these analyses, insulin levels were included both as a continuous variable and as a categorical variable (with Q1 as the reference group), and odds ratios (ORs) with 95% confidence intervals (CIs) were calculated. Model 1: unadjusted; Model 2: adjusted for age, gender, race/ethnicity, education level, marital status, and PIR; Model 3: further adjusted for BMI, smoking status, hypertension, diabetes, total cholesterol, triglycerides, AST, ALT, and hs-CRP based on Model 2. Third, RCS models were used to explore potential nonlinear associations between serum insulin levels and NAFLD prevalence. Fourth, subgroup analyses were performed to examine whether the association between serum insulin levels and NAFLD prevalence was consistent across different population characteristics. Stratification variables included age, gender, race/ethnicity, education level, marital status, PIR, BMI, hypertension, diabetes, and smoking status. Fifth, sensitivity analyses were performed to test the robustness of the findings. First, participants with any missing data were excluded to assess results in a complete-case dataset. Second, diabetic participants receiving insulin therapy, those taking insulin secretagogues, or both were excluded to minimize potential confounding by antidiabetic medications. Third, all diabetic participants, regardless of treatment type, were excluded in an additional analysis. Finally, participants with serum insulin levels in the top and bottom 2.5% were excluded to reduce the influence of extreme outliers. Sixth, to further evaluate the predictive ability of serum insulin for ultrasound-defined NAFLD, ROC curves were plotted, and the area under the curve (AUC) was calculated. ROC analysis was used to assess the sensitivity and specificity of insulin as a continuous variable in distinguishing NAFLD from non-NAFLD individuals. All analyses accounted for the complex NHANES sampling design, incorporating sample weights to ensure national representativeness. Data were analyzed using R (version 4.2.2; R Foundation for Statistical Computing, Vienna, Austria) and Free Statistics (version 1.9; Beijing Fengrui Colin Medical Technology Co., Ltd., Beijing, China). Statistical tests were two-sided, with *P* < .05 considered significant.

## 3. Results

### 3.1. Participant selection

**Figure 1.**
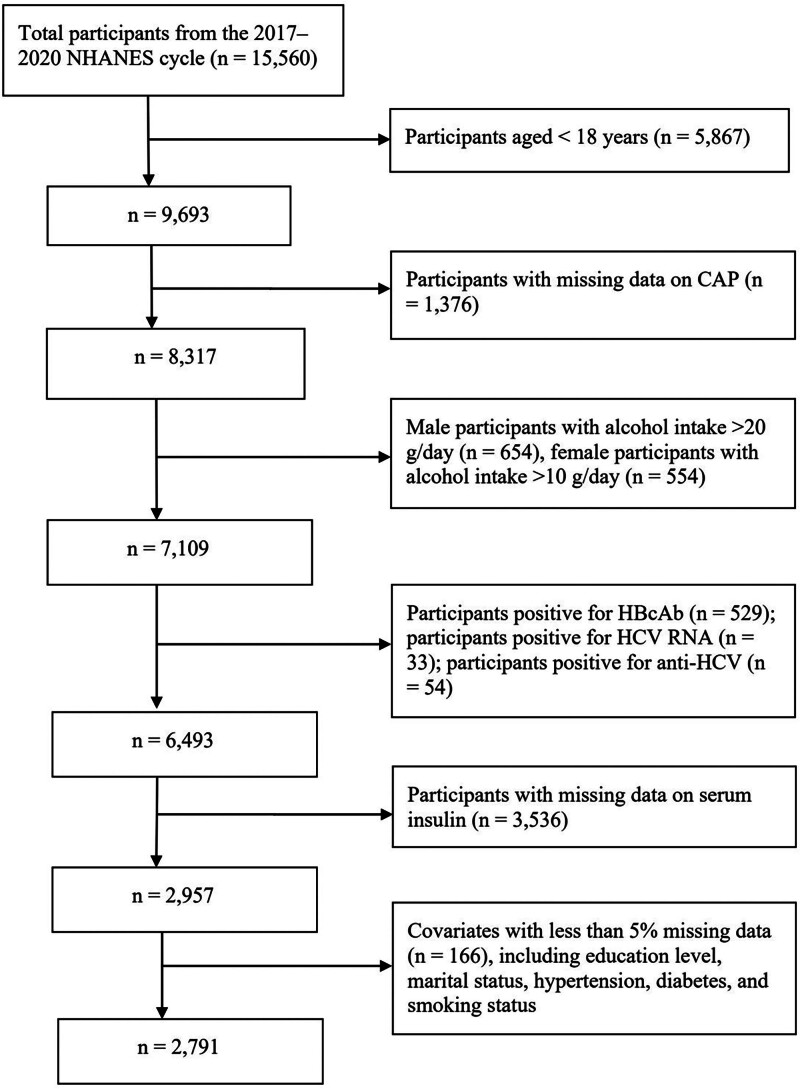
Overview of the study design and participant selection process. CAP = controlled attenuation parameter, HCV = hepatitis C virus, NHANES = National Health and Nutrition Examination Survey.

A total of 15,560 participants from the NHANES 2017–2020 cycle were initially considered for this study. First, individuals under 18 years of age (n = 5867) were excluded, leaving 9693 adult participants. Second, participants without available liver CAP measurements (n = 1376) were excluded, resulting in 8317 individuals. Third, those who exceeded the alcohol consumption limits (male > 20 g/d and female > 10 g/d) were excluded (n = 1208), leaving 7109 participants. Next, individuals with potential viral hepatitis infection risks were excluded, including those who were positive for hepatitis B core antibody (n = 529), HCV RNA (n = 33), or anti-HCV (n = 54), leaving 6493 participants. Participants missing serum insulin data (n = 3536) were further excluded, resulting in 2957 individuals. Finally, for covariates with <5% missing data (including education level, marital status, hypertension, diabetes, and smoking status), participants with missing values (n = 166) were excluded. A total of 2791 eligible participants were ultimately included in the final statistical analysis (Fig. 1).

### 3.2. Baseline characteristics of the study population

Table 1 summarizes the baseline characteristics of participants grouped by quartiles of serum insulin levels. A total of 2791 individuals were included, representing approximately 39.4 million United States adults after weighting. Participants were divided into 4 groups based on serum insulin levels: Q1 (0.71–6.41 μU/mL, n = 698) with a NAFLD prevalence of 27.1%; Q2 (6.44–10.59 μU/mL, n = 693) with a prevalence of 52.5%; Q3 (10.61–17.18 μU/mL, n = 702) with a prevalence of 71.2%; and Q4 (17.20–512.50 μU/mL, n = 698) with a prevalence increasing to 87.5%. The prevalence of NAFLD increased progressively with higher serum insulin levels (*P* < .001). In terms of sociodemographic and clinical characteristics, the proportion of Mexican American or other Hispanic participants was higher in the Q4 group. Participants in the Q4 group also had significantly higher BMI and higher rates of hypertension and diabetes compared to the other groups. Furthermore, serum levels of triglycerides, ALT, AST, and hs-CRP were markedly elevated in the higher insulin groups (Table 1).

**Table 1:** General characteristics of the study population.

Variables	Total (n = 2791)	Quartiles of serum insulin levels (μU/mL)				*P* value
		Q1 (0.71–6.41), n = 698	Q2 (6.44–10.59), n = 693	Q3 (10.61–17.18), n = 702	Q4 (17.20–512.50), n = 698	
Weighted sample size	39,402,901	10,258,496	10,305,768	9787,306	9051,331	
Ultrasound-defined NAFLD, n (%)						<.001
Absent	1127 (40.4)	509 (72.9)	329 (47.5)	202 (28.8)	87 (12.5)	
Present	1664 (59.6)	189 (27.1)	364 (52.5)	500 (71.2)	611 (87.5)	
Age (yr), Median (IQR)	51.0 (35.0–64.0)	47.5 (32.0–63.0)	52.0 (36.0–65.0)	53.0 (38.0–65.0)	50.5 (35.0–63.0)	.010
Gender, n (%)						.424
Male	1330 (47.7)	350 (50.1)	325 (46.9)	319 (45.4)	336 (48.1)	
Female	1461 (52.3)	348 (49.9)	368 (53.1)	383 (54.6)	362 (51.9)	
Race/ethnicity, n (%)						.002
Mexican American/Other Hispanic	712 (25.5)	147 (21.1)	170 (24.5)	182 (25.9)	213 (30.5)	
Non-Hispanic White/Non-Hispanic Black/Other Race	2079 (74.5)	551 (78.9)	523 (75.5)	520 (74.1)	485 (69.5)	
Education level, n (%)						.079
Less than high school	544 (19.5)	119 (17)	138 (19.9)	133 (18.9)	154 (22.1)	
High school or equivalent	672 (24.1)	171 (24.5)	167 (24.1)	157 (22.4)	177 (25.4)	
College or above	1575 (56.4)	408 (58.5)	388 (56)	412 (58.7)	367 (52.6)	
Marital status, n (%)						.144
Married/Living with partner	1638 (58.7)	383 (54.9)	405 (58.4)	434 (61.8)	416 (59.6)	
Widowed/Divorced/Separated	601 (21.5)	151 (21.6)	169 (24.4)	149 (21.2)	132 (18.9)	
Never married	552 (19.8)	164 (23.5)	119 (17.2)	119 (17)	150 (21.5)	
PIR, Median (IQR)	2.6 (1.4–3.7)	2.6 (1.4–4.0)	2.6 (1.5–3.6)	2.6 (1.4–4.0)	2.4 (1.2–3.2)	.061
BMI (kg/m^2^), Median (IQR)	29.1 (25.1–34.2)	24.2 (21.5–27.3)	28.0 (25.0–31.3)	30.1 (27.1–34.4)	34.9 (31.0–40.6)	<.001
Hypertension, n (%)						<.001
Absent	1741 (62.4)	520 (74.5)	461 (66.5)	394 (56.1)	366 (52.4)	
Present	1050 (37.6)	178 (25.5)	232 (33.5)	308 (43.9)	332 (47.6)	
Diabetes, n (%)						<.001
Nondiabetic	2315 (82.9)	627 (89.8)	597 (86.1)	570 (81.2)	521 (74.6)	
Diabetic	476 (17.1)	71 (10.2)	96 (13.9)	132 (18.8)	177 (25.4)	
Smoking status, n (%)						.121
Never smoker	1670 (59.8)	413 (59.2)	415 (59.9)	439 (62.5)	403 (57.7)	
Former smoker	665 (23.8)	142 (20.3)	166 (24)	167 (23.8)	190 (27.2)	
Current smoker	456 (16.3)	143 (20.5)	112 (16.2)	96 (13.7)	105 (15)	
Total cholesterol (mg/dL), Median (IQR)	179.0 (156.0–207.0)	179.0 (157.0–208.0)	179.0 (153.0–208.0)	181.0 (158.2–210.0)	177.0 (155.0–203.0)	.055
Triglycerides (mg/dL), Median (IQR)	91.0 (61.0–134.5)	63.0 (47.0–89.0)	82.0 (58.0–119.0)	107.5 (73.0–147.8)	121.0 (85.0–171.0)	<.001
AST (U/L), Median (IQR)	19.0 (15.0–23.0)	18.0 (16.0–22.0)	18.0 (15.0–22.0)	19.0 (15.0–23.0)	19.0 (16.0–26.0)	<.001
ALT (U/L), Median (IQR)	17.0 (13.0–25.0)	15.0 (11.0–19.8)	16.0 (12.0–21.0)	18.0 (13.0–26.0)	22.0 (16.0–33.0)	<.001
hs-CRP (mg/L), Median (IQR)	2.1 (0.9–4.5)	1.0 (0.5–2.7)	1.7 (0.8–3.7)	2.3 (1.1–4.4)	3.8 (1.7–7.7)	<.001

ALT = alanine aminotransferase, AST = aspartate aminotransferase, BMI = body mass index, hs-CRP = high-sensitivity C-reactive protein, IQR = interquartile range, NAFLD = nonalcoholic fatty liver disease, PIR = poverty income ratio.

### 3.3. Association between serum insulin levels and the prevalence of ultrasound-defined NAFLD

In logistic regression analysis, serum insulin levels (treated as a continuous variable) were significantly positively associated with ultrasound-defined NAFLD. In the unadjusted model (Model 1), the OR was 1.14 (95% CI: 1.10–1.18, *P* < .001). In Model 2, the OR was 1.15 (95% CI: 1.11–1.18, *P* < .001). After further adjustment in Model 3, the association remained significant (OR: 1.04, 95% CI: 1.01–1.07, *P* = .014). When using Q1 (0.71–6.41 μU/mL) as the reference group, the odds of having NAFLD increased progressively across Q2 (6.44–10.59 μU/mL), Q3 (10.61–17.18 μU/mL), and Q4 (17.20–512.50 μU/mL). In Model 3, the OR for Q2 was 1.92 (95% CI: 1.21–3.04, *P* = .017), for Q3 was 2.22 (95% CI: 1.15–4.29, *P* = .028), and for Q4 was 3.61 (95% CI: 1.84–7.08, *P* = .006). Trend analysis showed a significant linear increase in the odds of NAFLD across insulin quartiles (*P* for trend = .003; Table 2).

**Table 2:** Association between serum insulin and ultrasound-defined NAFLD across logistic regression models.

	Model 1		Model 2		Model 3	
	OR (95% CI)	*P*-value	OR (95% CI)	*P*-value	OR (95% CI)	*P*-value
Continuous	1.14 (1.10–1.18)	<.001	1.15 (1.11–1.18)	<.001	1.04 (1.01–1.07)	.014
Quartile						
Q1 (n = 698)	Reference		Reference		Reference	
Q2 (n = 693)	3.64 (2.37–5.60)	<.001	3.64 (2.46–5.38)	<.001	1.92 (1.21–3.04)	.017
Q3 (n = 702)	6.49 (4.43–9.51)	<.001	6.81 (4.52–10.26)	<.001	2.22 (1.15–4.29)	.028
Q4 (n = 698)	20.86 (11.43–38.07)	<.001	25.66 (14.56–45.23)	<.001	3.61 (1.84–7.08)	.006
*P* for trend		<.001		<.001		.003

Model 1: No covariates included. Model 2: Adjusted for demographic factors: age, gender, race/ethnicity, education level, marital status, and PIR. Model 3: Based on Model 2, additionally adjusted for BMI, smoking status, diabetes, hypertension, total cholesterol, triglycerides, AST, ALT, and hs-CRP.

ALT = alanine aminotransferase, AST = aspartate aminotransferase, BMI = body mass index, hs-CRP = high-sensitivity C-reactive protein, CI = confidence interval, NAFLD = nonalcoholic fatty liver disease, OR = odds ratio, PIR = poverty income ratio.

### 3.4. Results of RCS analysis

**Figure 2.**
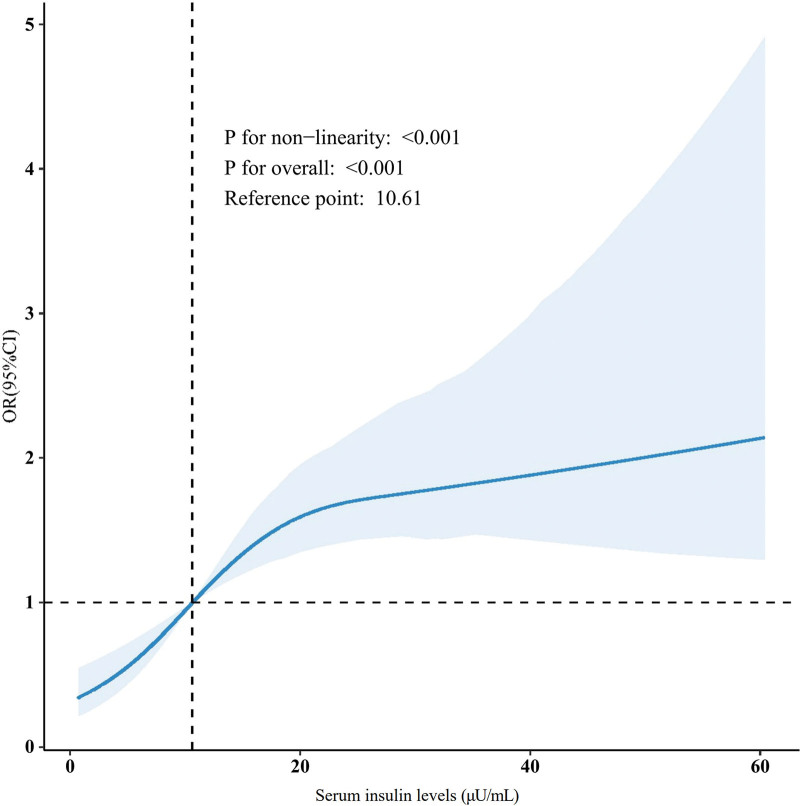
Multivariate-adjusted RCS analysis showing the association between serum insulin levels and ultrasound-defined NAFLD prevalence. CI = confidence interval, NAFLD = nonalcoholic fatty liver disease, OR = odds ratio, RCS = restricted cubic spline.

RCS analysis, adjusted for multiple covariates, showed a significant nonlinear positive association between serum insulin levels and the odds of having NAFLD (*P* for non-linearity < .001; Fig. 2).

### 3.5. Subgroup analyses and interaction assessment of the association between serum insulin levels and NAFLD prevalence

**Figure 3.**
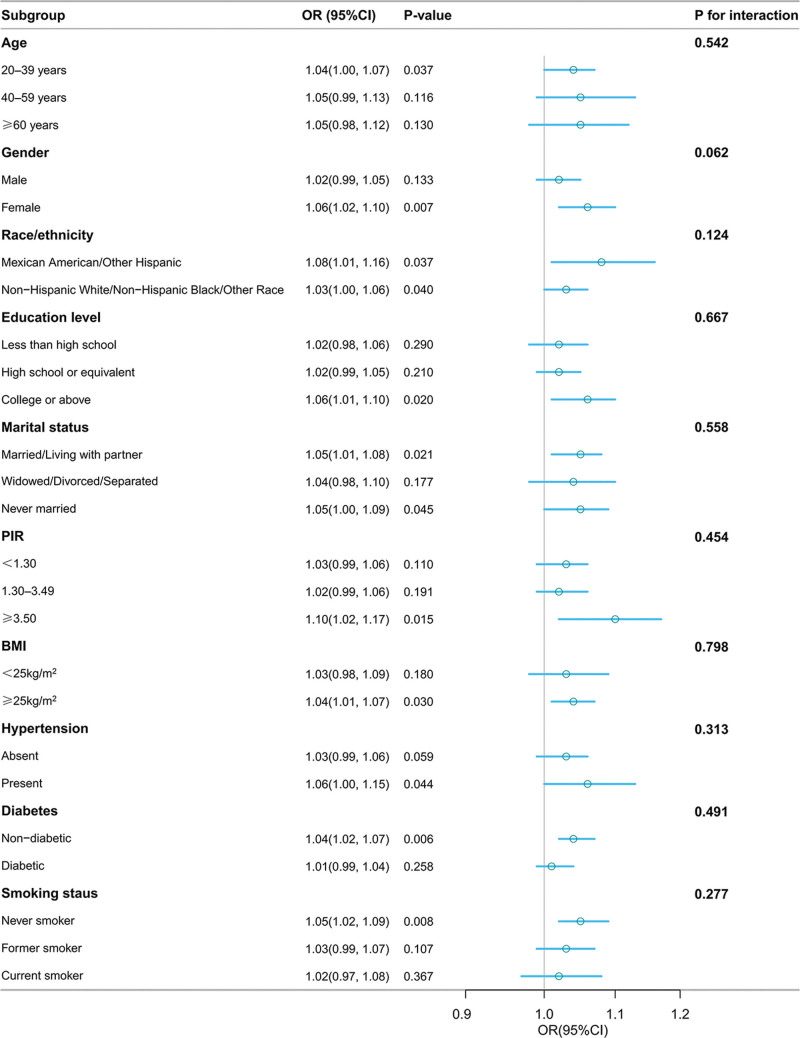
Subgroup analysis evaluating the association between serum insulin levels and ultrasound-defined NAFLD prevalence. BMI = body mass index, CI = confidence interval, NAFLD = nonalcoholic fatty liver disease, OR = odds ratio, PIR = poverty income ratio.

In subgroup analyses based on multivariable logistic regression models, the positive association between serum insulin levels and the prevalence of ultrasound-defined NAFLD remained consistent across multiple subgroups. Interaction analyses showed no significant interactions between serum insulin levels and the subgroup variables in relation to NAFLD (all *P* for interaction > .05), suggesting that the association was robust across different population subgroups (Fig. 3).

### 3.6. Results of sensitivity analyses

Multiple sensitivity analyses were conducted to verify the robustness of the findings. The positive association between serum insulin levels and the prevalence of NAFLD remained consistent across various sensitivity analyses, including exclusion of participants with missing data, those receiving insulin therapy, those taking insulin secretagogues, all diabetic participants, and those with extreme serum insulin values. The direction and significance of the association were largely unchanged, confirming the stability and robustness of the results (Table 3).

**Table 3:** Sensitivity analyses for the association between serum insulin and ultrasound-defined NAFLD.

Sensitivity analysis method	Model 1		Model 2		Model 3	
	OR (95% CI)	*P*-value	OR (95% CI)	*P*-value	OR (95% CI)	*P*-value
Analysis after excluding all missing data (n = 2369)	1.14 (1.10–1.18)	<.001	1.15 (1.11–1.19)	<.001	1.04 (1.00–1.08)	.045
Excluding diabetic participants on insulin therapy (n = 2663)	1.15 (1.11–1.18)	<.001	1.16 (1.12–1.19)	<.001	1.04 (1.02–1.07)	.008
Excluding diabetic participants on insulin secretagogues (n = 2658)	1.14 (1.10–1.18)	<.001	1.15 (1.11–1.18)	<.001	1.04 (1.02–1.07)	.015
Excluding participants using insulin or insulin secretagogues (n = 2551)	1.14 (1.10–1.18)	<.001	1.15 (1.12–1.19)	<.001	1.04 (1.02–1.07)	.008
Excluding all diabetic participants (n = 2315)	1.14 (1.10–1.18)	<.001	1.15 (1.12–1.19)	<.001	1.04 (1.02–1.07)	.006
Excluding top and bottom 2.5% of serum insulin levels (n = 2651)	1.15 (1.11–1.19)	<.001	1.16 (1.13–1.20)	<.001	1.05 (1.03–1.08)	.003

Model 1: No covariates included. Model 2: Adjusted for demographic factors: age, gender, race/ethnicity, education level, marital status, and PIR. Model 3: Based on Model 2, additionally adjusted for BMI, smoking status, diabetes, hypertension, total cholesterol, triglycerides, AST, ALT, and hs-CRP.

ALT = alanine aminotransferase, AST = aspartate aminotransferase, BMI = body mass index, hs-CRP = high-sensitivity C-reactive protein, CI = confidence interval, NAFLD = nonalcoholic fatty liver disease, OR = odds ratio, PIR = poverty income ratio.

### 3.7. Results of ROC curve analysis

**Figure 4.**
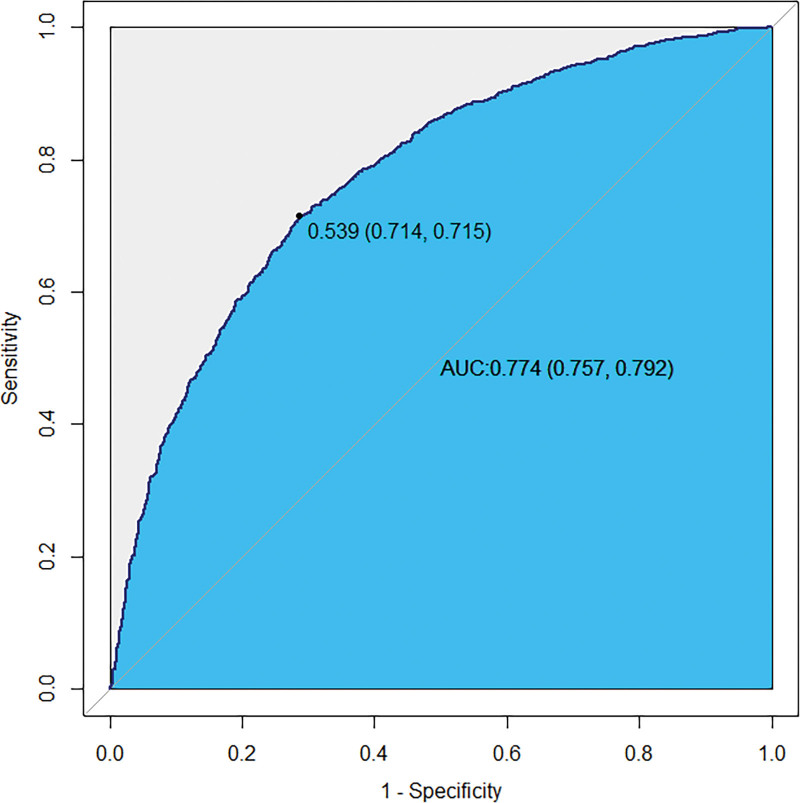
ROC curve for serum insulin levels in predicting ultrasound-defined NAFLD. AUC = area under the curve, NAFLD = nonalcoholic fatty liver disease, ROC = receiver operating characteristic.

To further evaluate the predictive ability of serum insulin levels alone for ultrasound-defined NAFLD, a ROC curve was plotted, and the AUC was calculated. The results showed that the AUC was 0.774 (95% CI: 0.757–0.792), indicating good discriminative ability of serum insulin for NAFLD. The optimal cutoff value identified from the ROC curve was 0.539, at which the model achieved a sensitivity of 0.714 and a specificity of 0.715 (Fig. 4).

## 4. Discussion

Based on nationally representative data from NHANES 2017–2020, this study utilized the CAP measured by liver transient elastography to perform a noninvasive and quantitative assessment of NAFLD. The association between serum insulin levels and NAFLD was systematically analyzed. The results showed that serum insulin levels were significantly positively associated with the prevalence of ultrasound-defined NAFLD. This association remained robust after adjusting for a range of demographic and clinical metabolic factors. As serum insulin levels increased, the prevalence of NAFLD significantly rose, showing a nonlinear dose-response relationship. In addition, serum insulin demonstrated good discriminative ability for predicting NAFLD, with an AUC of 0.774, further supporting its potential as a metabolic biomarker. Sensitivity analyses and subgroup analyses further confirmed the robustness and broad applicability of the findings.

Previous studies have suggested that IR and elevated serum insulin levels are closely associated with NAFLD. Bugianesi et al used a two-step hyperglycemic–hyperinsulinemic clamp technique, combined with isotope-labeled glucose and glycerol tracers and energy metabolism measurements, to compare 12 biopsy-confirmed NAFLD patients without obesity, dyslipidemia, or hyperglycemia with 6 healthy controls. The results showed that although hepatic glucose production was normal in NAFLD patients, peripheral glucose utilization was significantly impaired, accompanied by reductions in glucose oxidation and glycogen synthesis. In addition, NAFLD patients exhibited increased fasting lipolysis and fat oxidation, along with a reduced suppression of lipolysis under insulin stimulation. These findings suggest that inherent IR exists in NAFLD patients even in the absence of overt metabolic abnormalities.^[26]^ Salgado et al conducted a study including 116 patients diagnosed with NAFLD by clinical, biochemical, imaging, or biopsy criteria, and 88 healthy controls without liver disease and with normal oral glucose tolerance test results. All participants underwent oral glucose tolerance test and had blood samples collected to measure serum insulin levels using an immunofluorescence method. The results demonstrated that serum insulin levels were significantly higher in NAFLD patients compared to healthy controls.^[27]^

IR and the accompanying elevation in serum insulin levels play a critical role in the development and progression of NAFLD. The underlying mechanisms involve several aspects. First, during IR, the ability of insulin to suppress lipolysis in adipose tissue is impaired, leading to an increased release of free fatty acids (FFAs) into the circulation. These FFAs are transported to the liver, where they are re-esterified into triglycerides, promoting hepatic fat accumulation.^[28,29]^ Second, elevated FFAs in the liver not only enhance fat deposition but also induce mitochondrial dysfunction and lipotoxicity, further exacerbating hepatocellular injury and inflammation.^[30,31]^ Third, IR is associated with increased secretion of pro-inflammatory cytokines, such as tumor necrosis factor alpha and IL-6, from adipose tissue, leading to systemic low-grade chronic inflammation that aggravates hepatic inflammation and promotes NAFLD progression.^[32–34]^ Fourth, IR inhibits mitochondrial β-oxidation and enhances oxidative stress, resulting in excessive generation of reactive oxygen species, which further disrupts lipid metabolism and aggravates hepatic triglyceride accumulation and fat deposition.^[35]^ Finally, hyperinsulinemia can directly stimulate hepatic lipogenesis by enhancing de novo lipogenesis pathways, thereby worsening hepatic fat accumulation.^[36]^ These mechanisms collectively accelerate the development and progression of NAFLD.

When compared with previous studies, this study has several strengths. First, the data were derived from the nationally representative NHANES 2017–2020 survey, ensuring good representativeness and generalizability, which enhances the applicability of the study findings. Second, NAFLD was defined based on the CAP measured by transient elastography, providing a noninvasive and quantitative assessment of hepatic fat content. When compared with conventional ultrasound, this method offers higher sensitivity and specificity, improving the accuracy of NAFLD diagnosis. Third, a wide range of potential confounders, including demographic characteristics, metabolic indicators, and inflammatory markers, were comprehensively adjusted for in the statistical analyses, further strengthening the robustness of the results. Fourth, consistency and stability of the association between serum insulin levels and NAFLD prevalence were validated from multiple perspectives through RCS analysis, sensitivity analyses, and subgroup analyses, enhancing the credibility of the findings. Fifth, this study not only reveals the close association between serum insulin levels and ultrasound-quantified NAFLD prevalence from an epidemiological perspective but also provides important guidance for clinical practice, particularly for ultrasound departments. The results suggest that in routine fatty liver screening and diagnosis, clinical sonographers could incorporate patients’ serum insulin levels as an auxiliary reference marker to facilitate earlier metabolic risk assessment and intervention for individuals with suspicious imaging findings. Although the discriminative ability of serum insulin alone was moderate, this performance remains meaningful for clinical application, as NAFLD is a multifactorial metabolic disease and a single biomarker cannot fully capture all relevant determinants. Especially in patients whose CAP values are close to the diagnostic threshold, or those with mild imaging findings but elevated serum insulin levels, greater clinical vigilance is warranted. Such patients may have an underlying tendency toward metabolic abnormalities, and early lifestyle interventions or further metabolic evaluations should be recommended accordingly.

This study has a large sample size and multiple analytical strengths but also has certain limitations. First, due to the cross-sectional design, causal relationships between elevated serum insulin levels and the development of NAFLD cannot be established.

Second, the diagnosis of NAFLD was based on CAP values measured by transient elastography. Although this method is more accurate than conventional ultrasound, it cannot fully replace liver biopsy, especially in assessing inflammatory activity and the degree of fibrosis. Third, serum insulin levels may be influenced by short-term factors such as diet and stress. Although we included fasting measurements whenever possible, physiological fluctuations could still have introduced potential bias. Fourth, despite adjusting for multiple potential confounders, the inherent limitations of the NHANES database mean that residual confounding from unmeasured or uncontrolled variables cannot be completely ruled out. Fifth, the study population was predominantly from the United States, so the generalizability of the findings to other racial and regional populations requires further validation. Because the NHANES cohort represents a multi-ethnic United States population, genetic and metabolic differences may limit direct extrapolation to Chinese populations. Future studies in homogeneous Chinese cohorts are needed to verify these associations.

## 5. Conclusion

Based on nationally representative data from the NHANES 2017–2020 survey, this study found a significant positive association between serum insulin levels and the prevalence of ultrasound-defined NAFLD as measured by the CAP. This association remained robust across various subgroups and in multiple sensitivity analyses. Elevated serum insulin levels may serve as an important metabolic marker for the early identification of fatty liver, providing valuable supplementary information for the comprehensive interpretation of ultrasound screening results. This finding highlights the potential clinical value of incorporating serum insulin levels into the risk assessment and management of fatty liver disease. Future studies with longitudinal follow-up are warranted to explore the relationship between dynamic changes in serum insulin levels and quantitative ultrasound measures, which could provide stronger evidence for early intervention and comprehensive management of NAFLD.

Supplemental Digital Content “Table S1” is available for this article (https://links.lww.com/MD/R170).

## Author contributions

**Conceptualization:** Mao Li.

**Data curation:** Mao Li, Shunhua Qiu, Min Xiang.

**Formal analysis:** Shunhua Qiu.

**Methodology:** Mao Li.

**Validation:** Min Xiang.

**Visualization:** Shunhua Qiu.

**Writing – original draft:** Mao Li, Shunhua Qiu, Min Xiang.

**Writing – review & editing:** Mao Li, Shunhua Qiu, Min Xiang.

## Supplementary Material


